# (2*Z*)-3-(4-Chloro­anilino)-1-(5-hy­droxy-3-methyl-1-phenyl-1*H*-pyrazol-4-yl)but-2-en-1-one

**DOI:** 10.1107/S1600536811029473

**Published:** 2011-07-30

**Authors:** Abdullah M. Asiri, Abdulrahman O. Al-Youbi, Khalid A. Alamry, Hassan M. Faidallah, Seik Weng Ng, Edward R. T. Tiekink

**Affiliations:** a Chemistry Department, Faculty of Science, King Abdulaziz University, PO Box 80203, Jeddah, Saudi Arabia; b The Center of Excellence for Advanced Materials Research, King Abdulaziz University, Jeddah, PO Box 80203, Saudi Arabia; c Department of Chemistry, University of Malaya, 50603 Kuala Lumpur, Malaysia

## Abstract

With the exception of the terminal benzene rings, the atoms in the title compound, C_20_H_18_ClN_3_O_2_, are approximately coplanar (r.m.s. deviation = 0.0495 Å). The benzene/chloro­benzene rings form dihedral angles of 3.02 (4) and 41.59 (5)°, respectively, with this plane. The hy­droxy, amino and carbonyl groups all lie to the same side of the mol­ecule, enabling the formation of intra­molecular O—H⋯O and N—H⋯O hydrogen bonds that close *S*(6) rings. The configuration about the 2-butene bond is *Z*. Supra­molecular chains mediated by C—H⋯Cl inter­actions and aligned along the *c* axis are found in the crystal packing. These assemble into layers that are connected by weak π–π inter­actions between centrosymmetrically related chloro­benzene rings [3.8156 (9) Å].

## Related literature

For background to the synthesis, see: Gelin *et al.* (1983 ▶); Bendaas *et al.* (1999 ▶).
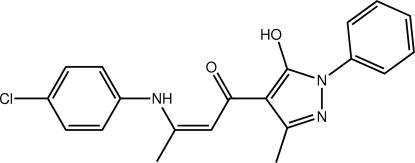


## Experimental

### 

#### Crystal data

C_20_H_18_ClN_3_O_2_
*M* *_r_* = 367.82
Monoclinic, 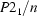

*a* = 10.7782 (3) Å
*b* = 12.6349 (4) Å
*c* = 12.9071 (4) Å
β = 100.956 (3)°
*V* = 1725.67 (9) Å^3^
*Z* = 4
Mo *K*α radiation
μ = 0.24 mm^−1^
*T* = 100 K
0.30 × 0.25 × 0.20 mm

#### Data collection

Agilent SuperNova Dual diffractometer with an Atlas detector
Absorption correction: multi-scan (*CrysAlis PRO*; Agilent, 2010 ▶) *T*_min_ = 0.931, *T*_max_ = 0.953
8785 measured reflections
3860 independent reflections
3199 reflections with *I* > 2σ(*I*)
*R*_int_ = 0.025

#### Refinement

*R*[*F* ^2^ > 2σ(*F* ^2^)] = 0.039
*wR*(*F* ^2^) = 0.103
*S* = 1.01
3860 reflections
245 parameters
H atoms treated by a mixture of independent and constrained refinement
Δρ_max_ = 0.27 e Å^−3^
Δρ_min_ = −0.30 e Å^−3^

### 

Data collection: *CrysAlis PRO* (Agilent, 2010 ▶); cell refinement: *CrysAlis PRO*; data reduction: *CrysAlis PRO*; program(s) used to solve structure: *SHELXS97* (Sheldrick, 2008 ▶); program(s) used to refine structure: *SHELXL97* (Sheldrick, 2008 ▶); molecular graphics: *ORTEP-3* (Farrugia, 1997 ▶) and *DIAMOND* (Brandenburg, 2006 ▶); software used to prepare material for publication: *publCIF* (Westrip, 2010 ▶).

## Supplementary Material

Crystal structure: contains datablock(s) global, I. DOI: 10.1107/S1600536811029473/hg5069sup1.cif

Structure factors: contains datablock(s) I. DOI: 10.1107/S1600536811029473/hg5069Isup2.hkl

Supplementary material file. DOI: 10.1107/S1600536811029473/hg5069Isup3.cml

Additional supplementary materials:  crystallographic information; 3D view; checkCIF report

## Figures and Tables

**Table 1:** Hydrogen-bond geometry (Å, °)

*D*—H⋯*A*	*D*—H	H⋯*A*	*D*⋯*A*	*D*—H⋯*A*
O1—H1⋯O2	0.96 (3)	1.64 (3)	2.5283 (16)	153 (3)
N3—H3⋯O2	0.91 (2)	1.93 (2)	2.6678 (18)	136.9 (18)
C4—H4⋯Cl1^i^	0.95	2.81	3.6217 (18)	144

Symmetry code: (i) 
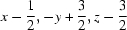
.

**Fig. 1.**
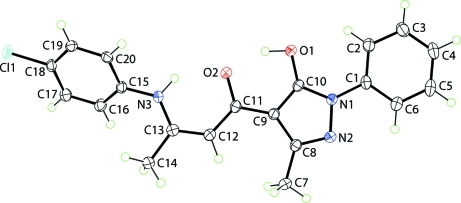
The molecular structure of (I) showing the atom-labelling scheme and displacement ellipsoids at the 50% probability level.

**Fig. 2.**
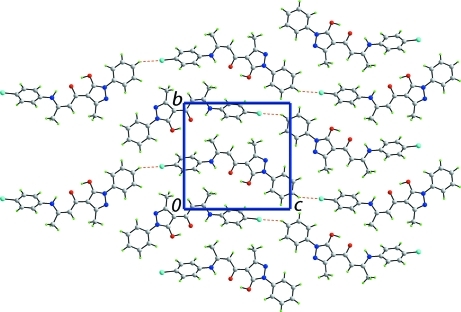
Assembly of supramolecular chains aligned along the c axis in (I) mediated by C—H···Cl interactions shown as orange dashed lines.

## supplementary crystallographic information

### Comment

The title compound (I) was isolated during an investigation of reactions between pyrazoles and aniline derivatives following literature precedents (Gelin *et al.*, 1983; Bendaas *et al.*, 1999). The molecular structure of (I), Fig. 1, features a *Z* configuration about the C12—C13 [1.376 (2) Å] bond. The hydroxy and amino groups are *syn* to the central carbonyl group and each forms a hydrogen bond to close a *S*(6) ring (Table 1). A result of this feature of the structure is that the central residue is planar; the values of the C10—C9—C11–O2, C9—C11—C12—C13 and C11—C12—C13—N3 torsion angles are 2.5 (2), -177.93 (14) and -0.4 (2) °, respectively. Indeed, the r.m.s. deviation for the non-hydrogen atoms comprising the entire molecule excluding the terminal benzene rings is 0.0495 Å. The benzene and chlorobenzene rings form dihedral angles of 3.02 (4) and 41.59 (5) °, respectively, with the central plane.

The most prominent feature of the crystal packing is the formation of supramolecular chains mediated by C—H···Cl interactions, Table 1. The chains assemble into layers in the *bc* plane, Fig. 2. The closest interactions between layers stacking along the *a* direction are weak π–π contacts between centrosymmetrically related chlorobenzene rings [3.8156 (9) Å for symmetry operation: 2 - *x*, 1 - *y*, 2 - *z*].

### Experimental

A solution of 4-acetoacetyl-5-hydroxy-3-methyl-1-*p*-sulfamylphenypyrazole (1.7 g, 0.005 mol) and 4-chloroaniline (0.63 g, 0.005 mole) in ethanol (25 ml) was refluxed for 2 h. The precipitate, obtained from the hot solution, was collected, washed with methanol, and recrystallized from ethanol-benzene as orange crystals; *M*.pt 505–507 K.

### Refinement

Carbon-bound H-atoms were placed in calculated positions [C—H 0.95 to 0.98 Å, *U*_iso_(H) 1.2 to 1.5*U*_eq_(C)] and were included in the refinement in the riding model approximation. The hydroxy- and amino- H-atoms were located in a difference Fourier map, and subsequently refined freely.

### Crystal data

**Table:**

C_20_H_18_ClN_3_O_2_	*F*(000) = 768
*M**_r_* = 367.82	*D*_x_ = 1.416 Mg m^−^^3^
Monoclinic, *P*2_1_/*n*	Mo *K*α radiation, λ = 0.71073 Å
Hall symbol: -P 2yn	Cell parameters from 4068 reflections
*a* = 10.7782 (3) Å	θ = 2.5–29.3°
*b* = 12.6349 (4) Å	µ = 0.24 mm^−^^1^
*c* = 12.9071 (4) Å	*T* = 100 K
β = 100.956 (3)°	Block, orange
*V* = 1725.67 (9) Å^3^	0.30 × 0.25 × 0.20 mm
*Z* = 4	

### Data collection

**Table:**

Agilent SuperNova Dual diffractometer with an Atlas detector	3860 independent reflections
Radiation source: SuperNova (Mo) X-ray Source	3199 reflections with *I* > 2σ(*I*)
Mirror	*R*_int_ = 0.025
Detector resolution: 10.4041 pixels mm^-1^	θ_max_ = 27.5°, θ_min_ = 2.5°
ω scans	*h* = −13→11
Absorption correction: multi-scan (*CrysAlis PRO*; Agilent, 2010)	*k* = −13→16
*T*_min_ = 0.931, *T*_max_ = 0.953	*l* = −15→16
8785 measured reflections	

### Refinement

**Table:**

Refinement on *F*^2^	Primary atom site location: structure-invariant direct methods
Least-squares matrix: full	Secondary atom site location: difference Fourier map
*R*[*F*^2^ > 2σ(*F*^2^)] = 0.039	Hydrogen site location: inferred from neighbouring sites
*wR*(*F*^2^) = 0.103	H atoms treated by a mixture of independent and constrained refinement
*S* = 1.01	*w* = 1/[σ^2^(*F*_o_^2^) + (0.0495*P*)^2^ + 0.5858*P*] where *P* = (*F*_o_^2^ + 2*F*_c_^2^)/3
3860 reflections	(Δ/σ)_max_ < 0.001
245 parameters	Δρ_max_ = 0.27 e Å^−^^3^
0 restraints	Δρ_min_ = −0.30 e Å^−^^3^

### Special details

**Table:**

Geometry. All e.s.d.'s (except the e.s.d. in the dihedral angle between two l.s. planes) are estimated using the full covariance matrix. The cell e.s.d.'s are taken into account individually in the estimation of e.s.d.'s in distances, angles and torsion angles; correlations between e.s.d.'s in cell parameters are only used when they are defined by crystal symmetry. An approximate (isotropic) treatment of cell e.s.d.'s is used for estimating e.s.d.'s involving l.s. planes.
Refinement. Refinement of *F*^2^ against ALL reflections. The weighted *R*-factor *wR* and goodness of fit *S* are based on *F*^2^, conventional *R*-factors *R* are based on *F*, with *F* set to zero for negative *F*^2^. The threshold expression of *F*^2^ > σ(*F*^2^) is used only for calculating *R*-factors(gt) *etc*. and is not relevant to the choice of reflections for refinement. *R*-factors based on *F*^2^ are statistically about twice as large as those based on *F*, and *R*- factors based on ALL data will be even larger.

### Fractional atomic coordinates and isotropic or equivalent isotropic displacement parameters (Å^2^)

**Table:**

	*x*	*y*	*z*	*U*_iso_*/*U*_eq_	
Cl1	0.91036 (4)	0.59737 (3)	1.20821 (3)	0.02517 (13)	
O1	0.69340 (10)	0.75724 (9)	0.38953 (9)	0.0208 (3)	
O2	0.73186 (10)	0.65748 (9)	0.56249 (9)	0.0216 (3)	
N1	0.59886 (11)	0.64664 (11)	0.24798 (10)	0.0184 (3)	
N2	0.56236 (12)	0.54063 (11)	0.23365 (10)	0.0201 (3)	
N3	0.77747 (12)	0.55053 (12)	0.74398 (11)	0.0200 (3)	
C1	0.57853 (13)	0.71508 (14)	0.15919 (12)	0.0195 (3)	
C2	0.60600 (15)	0.82241 (14)	0.17012 (13)	0.0238 (4)	
H2	0.6377	0.8518	0.2377	0.029*	
C3	0.58657 (16)	0.88614 (15)	0.08098 (14)	0.0290 (4)	
H3A	0.6056	0.9595	0.0878	0.035*	
C4	0.53986 (16)	0.84422 (16)	−0.01787 (14)	0.0291 (4)	
H4	0.5276	0.8885	−0.0785	0.035*	
C5	0.51121 (15)	0.73772 (16)	−0.02783 (13)	0.0282 (4)	
H5	0.4780	0.7090	−0.0954	0.034*	
C6	0.53058 (14)	0.67263 (15)	0.06000 (13)	0.0236 (4)	
H6	0.5113	0.5993	0.0527	0.028*	
C7	0.56011 (16)	0.38241 (13)	0.33884 (14)	0.0241 (4)	
H7A	0.5145	0.3555	0.2710	0.036*	
H7B	0.5070	0.3749	0.3922	0.036*	
H7C	0.6383	0.3420	0.3606	0.036*	
C8	0.59131 (13)	0.49623 (13)	0.32786 (12)	0.0193 (3)	
C9	0.64770 (14)	0.57033 (13)	0.40580 (12)	0.0185 (3)	
C10	0.64989 (13)	0.66471 (13)	0.35046 (12)	0.0175 (3)	
C11	0.69441 (14)	0.56817 (13)	0.51866 (12)	0.0189 (3)	
C12	0.69884 (14)	0.47308 (13)	0.57726 (13)	0.0210 (3)	
H12	0.6726	0.4100	0.5393	0.025*	
C13	0.73809 (14)	0.46454 (13)	0.68479 (13)	0.0196 (3)	
C14	0.74065 (15)	0.35747 (13)	0.73563 (13)	0.0223 (4)	
H14A	0.7539	0.3030	0.6848	0.033*	
H14B	0.6601	0.3448	0.7581	0.033*	
H14C	0.8097	0.3548	0.7971	0.033*	
C15	0.80969 (14)	0.55777 (13)	0.85538 (12)	0.0187 (3)	
C16	0.73868 (14)	0.50806 (14)	0.92039 (13)	0.0223 (4)	
H16	0.6675	0.4665	0.8902	0.027*	
C17	0.77113 (14)	0.51871 (14)	1.02897 (13)	0.0220 (4)	
H17	0.7244	0.4827	1.0736	0.026*	
C18	0.87226 (14)	0.58229 (13)	1.07155 (12)	0.0192 (3)	
C19	0.94267 (14)	0.63411 (13)	1.00821 (13)	0.0199 (3)	
H19	1.0115	0.6781	1.0386	0.024*	
C20	0.91164 (14)	0.62108 (13)	0.89951 (13)	0.0192 (3)	
H20	0.9601	0.6555	0.8552	0.023*	
H1	0.721 (3)	0.739 (2)	0.462 (2)	0.077 (9)*	
H3	0.7859 (18)	0.6100 (17)	0.7060 (17)	0.035 (6)*	

### Atomic displacement parameters (Å^2^)

**Table:**

	*U*^11^	*U*^22^	*U*^33^	*U*^12^	*U*^13^	*U*^23^
Cl1	0.0307 (2)	0.0284 (2)	0.0149 (2)	0.00130 (16)	0.00068 (15)	−0.00084 (17)
O1	0.0259 (6)	0.0178 (6)	0.0170 (6)	−0.0017 (4)	0.0000 (4)	−0.0009 (5)
O2	0.0283 (6)	0.0188 (6)	0.0167 (6)	−0.0025 (5)	0.0019 (4)	−0.0017 (5)
N1	0.0190 (6)	0.0191 (7)	0.0162 (7)	−0.0017 (5)	0.0013 (5)	−0.0003 (6)
N2	0.0225 (7)	0.0184 (7)	0.0186 (7)	−0.0019 (5)	0.0019 (5)	−0.0015 (6)
N3	0.0245 (7)	0.0193 (7)	0.0150 (7)	−0.0012 (5)	0.0009 (5)	0.0018 (6)
C1	0.0151 (7)	0.0269 (9)	0.0164 (8)	0.0016 (6)	0.0031 (6)	0.0032 (7)
C2	0.0229 (8)	0.0279 (10)	0.0200 (8)	−0.0024 (7)	0.0029 (6)	0.0012 (7)
C3	0.0313 (9)	0.0291 (10)	0.0264 (9)	−0.0025 (7)	0.0051 (7)	0.0066 (8)
C4	0.0290 (9)	0.0385 (11)	0.0195 (9)	0.0003 (8)	0.0039 (7)	0.0096 (8)
C5	0.0255 (8)	0.0433 (12)	0.0152 (8)	0.0010 (8)	0.0021 (6)	0.0013 (8)
C6	0.0226 (8)	0.0293 (10)	0.0187 (8)	−0.0006 (7)	0.0037 (6)	−0.0018 (7)
C7	0.0285 (8)	0.0201 (9)	0.0226 (9)	−0.0031 (7)	0.0019 (7)	−0.0038 (7)
C8	0.0187 (7)	0.0207 (9)	0.0185 (8)	0.0010 (6)	0.0033 (6)	−0.0015 (7)
C9	0.0195 (7)	0.0190 (8)	0.0169 (8)	0.0004 (6)	0.0030 (6)	−0.0008 (7)
C10	0.0152 (7)	0.0204 (8)	0.0167 (8)	0.0003 (6)	0.0023 (6)	−0.0022 (7)
C11	0.0187 (7)	0.0215 (8)	0.0166 (8)	0.0008 (6)	0.0043 (6)	−0.0016 (7)
C12	0.0236 (8)	0.0200 (9)	0.0190 (8)	0.0001 (6)	0.0032 (6)	−0.0017 (7)
C13	0.0173 (7)	0.0202 (8)	0.0214 (8)	0.0005 (6)	0.0037 (6)	−0.0007 (7)
C14	0.0244 (8)	0.0200 (9)	0.0210 (9)	−0.0009 (6)	0.0009 (6)	0.0010 (7)
C15	0.0206 (7)	0.0187 (8)	0.0156 (8)	0.0036 (6)	0.0006 (6)	−0.0001 (6)
C16	0.0189 (7)	0.0261 (9)	0.0209 (8)	−0.0038 (6)	0.0012 (6)	−0.0004 (7)
C17	0.0229 (8)	0.0239 (9)	0.0198 (8)	0.0002 (6)	0.0052 (6)	0.0016 (7)
C18	0.0221 (7)	0.0195 (8)	0.0145 (8)	0.0052 (6)	−0.0002 (6)	0.0007 (6)
C19	0.0212 (8)	0.0164 (8)	0.0204 (8)	0.0008 (6)	0.0000 (6)	−0.0015 (7)
C20	0.0225 (8)	0.0155 (8)	0.0196 (8)	0.0018 (6)	0.0041 (6)	0.0016 (6)

### Geometric parameters (Å, °)

**Table:**

Cl1—C18	1.7438 (16)	C7—H7A	0.9800
O1—C10	1.3233 (19)	C7—H7B	0.9800
O1—H1	0.96 (3)	C7—H7C	0.9800
O2—C11	1.2921 (19)	C8—C9	1.423 (2)
N1—C10	1.3519 (19)	C9—C10	1.392 (2)
N1—N2	1.3984 (19)	C9—C11	1.448 (2)
N1—C1	1.419 (2)	C11—C12	1.416 (2)
N2—C8	1.321 (2)	C12—C13	1.376 (2)
N3—C13	1.349 (2)	C12—H12	0.9500
N3—C15	1.417 (2)	C13—C14	1.502 (2)
N3—H3	0.91 (2)	C14—H14A	0.9800
C1—C2	1.389 (2)	C14—H14B	0.9800
C1—C6	1.394 (2)	C14—H14C	0.9800
C2—C3	1.387 (2)	C15—C20	1.391 (2)
C2—H2	0.9500	C15—C16	1.389 (2)
C3—C4	1.385 (3)	C16—C17	1.385 (2)
C3—H3A	0.9500	C16—H16	0.9500
C4—C5	1.381 (3)	C17—C18	1.381 (2)
C4—H4	0.9500	C17—H17	0.9500
C5—C6	1.384 (2)	C18—C19	1.382 (2)
C5—H5	0.9500	C19—C20	1.389 (2)
C6—H6	0.9500	C19—H19	0.9500
C7—C8	1.490 (2)	C20—H20	0.9500
			
C10—O1—H1	100.3 (17)	C8—C9—C11	135.94 (15)
C10—N1—N2	110.06 (13)	O1—C10—N1	124.81 (14)
C10—N1—C1	131.22 (14)	O1—C10—C9	126.90 (14)
N2—N1—C1	118.72 (13)	N1—C10—C9	108.29 (14)
C8—N2—N1	105.88 (12)	O2—C11—C12	122.09 (14)
C13—N3—C15	128.07 (15)	O2—C11—C9	116.35 (14)
C13—N3—H3	114.3 (13)	C12—C11—C9	121.56 (15)
C15—N3—H3	117.6 (13)	C13—C12—C11	125.31 (16)
C2—C1—C6	120.24 (15)	C13—C12—H12	117.3
C2—C1—N1	121.03 (15)	C11—C12—H12	117.3
C6—C1—N1	118.73 (15)	N3—C13—C12	120.74 (15)
C1—C2—C3	119.12 (16)	N3—C13—C14	120.02 (14)
C1—C2—H2	120.4	C12—C13—C14	119.21 (15)
C3—C2—H2	120.4	C13—C14—H14A	109.5
C4—C3—C2	120.88 (18)	C13—C14—H14B	109.5
C4—C3—H3A	119.6	H14A—C14—H14B	109.5
C2—C3—H3A	119.6	C13—C14—H14C	109.5
C5—C4—C3	119.61 (17)	H14A—C14—H14C	109.5
C5—C4—H4	120.2	H14B—C14—H14C	109.5
C3—C4—H4	120.2	C20—C15—C16	119.74 (15)
C4—C5—C6	120.43 (17)	C20—C15—N3	118.36 (14)
C4—C5—H5	119.8	C16—C15—N3	121.82 (14)
C6—C5—H5	119.8	C17—C16—C15	120.35 (15)
C5—C6—C1	119.71 (17)	C17—C16—H16	119.8
C5—C6—H6	120.1	C15—C16—H16	119.8
C1—C6—H6	120.1	C18—C17—C16	119.18 (15)
C8—C7—H7A	109.5	C18—C17—H17	120.4
C8—C7—H7B	109.5	C16—C17—H17	120.4
H7A—C7—H7B	109.5	C19—C18—C17	121.39 (15)
C8—C7—H7C	109.5	C19—C18—Cl1	119.77 (12)
H7A—C7—H7C	109.5	C17—C18—Cl1	118.84 (13)
H7B—C7—H7C	109.5	C18—C19—C20	119.19 (15)
N2—C8—C9	111.44 (14)	C18—C19—H19	120.4
N2—C8—C7	118.63 (14)	C20—C19—H19	120.4
C9—C8—C7	129.91 (15)	C19—C20—C15	120.12 (15)
C10—C9—C8	104.32 (14)	C19—C20—H20	119.9
C10—C9—C11	119.70 (15)	C15—C20—H20	119.9
			
C10—N1—N2—C8	0.30 (16)	C11—C9—C10—O1	1.3 (2)
C1—N1—N2—C8	−179.71 (12)	C8—C9—C10—N1	−0.15 (16)
C10—N1—C1—C2	−3.9 (2)	C11—C9—C10—N1	−178.50 (13)
N2—N1—C1—C2	176.08 (13)	C10—C9—C11—O2	2.5 (2)
C10—N1—C1—C6	175.87 (14)	C8—C9—C11—O2	−175.18 (16)
N2—N1—C1—C6	−4.1 (2)	C10—C9—C11—C12	−177.32 (14)
C6—C1—C2—C3	−0.8 (2)	C8—C9—C11—C12	5.0 (3)
N1—C1—C2—C3	178.97 (14)	O2—C11—C12—C13	2.2 (2)
C1—C2—C3—C4	0.3 (2)	C9—C11—C12—C13	−177.93 (14)
C2—C3—C4—C5	0.5 (3)	C15—N3—C13—C12	173.70 (14)
C3—C4—C5—C6	−0.9 (3)	C15—N3—C13—C14	−8.2 (2)
C4—C5—C6—C1	0.4 (2)	C11—C12—C13—N3	−0.4 (2)
C2—C1—C6—C5	0.4 (2)	C11—C12—C13—C14	−178.47 (14)
N1—C1—C6—C5	−179.35 (14)	C13—N3—C15—C20	141.37 (16)
N1—N2—C8—C9	−0.40 (16)	C13—N3—C15—C16	−42.0 (2)
N1—N2—C8—C7	178.35 (13)	C20—C15—C16—C17	−1.9 (2)
N2—C8—C9—C10	0.35 (17)	N3—C15—C16—C17	−178.53 (15)
C7—C8—C9—C10	−178.22 (15)	C15—C16—C17—C18	2.2 (2)
N2—C8—C9—C11	178.29 (16)	C16—C17—C18—C19	−1.0 (2)
C7—C8—C9—C11	−0.3 (3)	C16—C17—C18—Cl1	178.69 (12)
N2—N1—C10—O1	−179.86 (13)	C17—C18—C19—C20	−0.6 (2)
C1—N1—C10—O1	0.1 (2)	Cl1—C18—C19—C20	179.74 (12)
N2—N1—C10—C9	−0.08 (16)	C18—C19—C20—C15	0.9 (2)
C1—N1—C10—C9	179.92 (14)	C16—C15—C20—C19	0.3 (2)
C8—C9—C10—O1	179.62 (14)	N3—C15—C20—C19	177.05 (14)

### Hydrogen-bond geometry (Å, °)

**Table:**

*D*—H···*A*	*D*—H	H···*A*	*D*···*A*	*D*—H···*A*
O1—H1···O2	0.96 (3)	1.64 (3)	2.5283 (16)	153 (3)
N3—H3···O2	0.91 (2)	1.93 (2)	2.6678 (18)	136.9 (18)
C4—H4···Cl1^i^	0.95	2.81	3.6217 (18)	144

Symmetry codes: (i) *x*−1/2, −*y*+3/2, *z*−3/2.
